# FOXC2 is a prognostic biomarker and contributes to the growth and invasion of human hepatocellular carcinoma

**DOI:** 10.1186/s12935-020-01265-0

**Published:** 2020-05-26

**Authors:** Jinzhang Chen, Xiaoxiang Rong, Xinhui Liu, Dayong Zheng, Xiaodong Rong, Fengsheng Chen, Peng Zhao, Feiye Liu, Jian Ruan

**Affiliations:** 1 State Key Laboratory of Organ Failure Research, Guangdong Provincial Key Laboratory of Viral Hepatitis Research, Department of Hepatology Unit and Infectious Diseases, Nanfang Hospital, Southern Medical University, Guangzhou, 510000 People’s Republic of China; 2 Department of Oncology, Nanfang Hospital, Southern Medical University, Guangzhou, 510000 Guangdong People’s Republic of China; 3 Cancer Center, Integrated Hospital of Traditional Chinese Medicine, Southern Medical University, Guangzhou, 510515 Guangdong People’s Republic of China; 4 Department of Radiation Oncology, Sun Yat-sen University Cancer Center, Sun Yat-Sen University, Guangzhou, 510515 Guangdong People’s Republic of China; 5 Department of Medical Oncology, The First Affiliated Hospital, School of Medicine, Zhejiang University, Hangzhou, 310003 Zhejiang Province, People’s Republic of China

**Keywords:** FOXC2, Ang-2, Hepatocellular carcinoma, Growth, Invasion

## Abstract

**Background:**

Forkhead box C2 (FOXC2) is a crucial factor involving in various cancers. However, its functions in hepatocellular carcinoma (HCC) is unknown. Here, we explored the role of FOXC2 in the progression of HCC and its potential mechanisms.

**Methods:**

FOXC2 expression in HCC tissue and cells were detected by immunohistochemistry or western blot and real-time PCR. CCK8, wound healing and transwell assay were used to measure cell growth and invasion. Tumor formation experiment was carried out to assess the tumorigenicity of HCC cells. Regulation of FOXC2 on Ang-2 was validated by luciferase assay and complementary experiments.

**Results:**

Increased FOXC2 expression was found to be associated positively with more aggressive clinicopathologic features. HCC patients with higher FOXC2 expression had significantly shorter overall survival. FOXC2 expression was indentified as an independent risk factor for resectable HCC. Increased FOXC2 expression accelerated the migration and invasion of HCC cells, accompanied by enhanced Ang-2 expression. Likewise, FOXC2 knockdown yielded opposite results. Moreover, FOXC2 stimulated the activation of the Ang-2 promoter. Suppression of Ang-2 expression hindered the FOXC2-mediated EMT processs, cell migration and invasion of HCC.

**Conclusions:**

FOXC2 is a novel prognostic predictor for HCC and may facilitate the growth and invasion through Ang-2.

## Background

As a fatal cancer with poor prognosis, hepatocellular carcinoma (HCC) causes the third-highest number of cancer-related mortality in sub-Saharan Africa, East Asia and the second-highest number deaths for males in China, with an increasing number of cases in Europe and the United States [1–3]. In the initial stages of HCC, the standard choice of treatment is the surgical resection in patients, although, 60–70% of patients still develop recurrence and metastasis within five years post-surgery [4]. Before the operation on the patients, poor prognosis occurs due to several clinicopathological features (e.g., large tumor size, poorly differentiated phenotype, poorly differentiated phenotype, and portal venous invasion), the molecular mechanisms guiding the development and pathogenesis of HCC remain to be elucidated.

A range of processes occurring in the cell, like metabolism, stress resistance, cell cycle arrest, apoptosis, and aging are regulated by the FOX (Forkhead box) family of proteins, which characteristically possess a DNA binding domain, that is evolutionarily conserved [5–7]. The FOXC2 is a type of FOX transcription factor, and its increased expression is an independent prognostic factor in several cancers, including NSCLC (non-small-cell lung cancer), cancer of breast, esophagus, colorectal, and that of the stomach [8–12]. FOXC2 may have a crucial role in the drug-resistant cancer phenotypes, like that of ovarian cancer, nasopharyngeal carcinoma, breast cancer, and osteosarcoma [13–16]. Moreover, enhanced expression of FOXC2 has been observed in the HCC tissues and relates inversely with patient survival [17], but data regarding the underlying role of FOXC2 in HCC development and progression are limited.

Abnormal angiogenesis is one of the hallmarks of malignancy and a research hotspot for the development of novel targeted regimen [18]. The A ngiopoietin-TIE2 pathway is key to the malignant angiogenesis, in which angiopoietin 1(Ang-1) and Ang-2 carried out opposite effects for the regulation of tumor vascularity. Mainly located in mesenchymal cells, Ang-1 facilitates vessel normalization by interacts with Tie-2 expressing in endothelial cells [19]. In contrast, Ang-2 performs as an antagonist with binding to Tie-2 or agonist to promote occurrence of tumor angiogenesis [20]. Increased expression of Ang-2 has been shown in both cancerous tissue and plasma of HCC patients [21, 22]. It is connected with more aggressive phenotype and unfavorable clinical outcomes [23]. By using the prediction tool, we identified Ang-2 as a possible downstream target of FOXC2, which led us to presume that FOXC2 may accelerate the development of HCC through regulating Ang-2.

Therefore, the expression levels and prognostic significance of FOXC2 in HCC tissues was determined in the present study. Additionally,we explored potential FOXC2 target genes involved in cell invasion and migration. Luciferase assays and western blot analysis were used to identify Ang-2 as a target gene of FOXC2. Ultimately, we knocked down endogenous Ang-2, using its shRNA, to elucidate the role of FOXC2 in tumor development.

## Methods

### Patients and samples

Paraffin-embedded HCC samples were obtained from the Nanfang hospital biobank. 280 HCC patients treated at Nanfang hospital between January 2009 and December 2014 were included. The inclusion criteria were (1) histologically confirmed the diagnosis and (2) no previous treatment. The exclusion criteria were (1) serious complications, (2) presence of other malignant diseases, or (3) incomplete follow-up data. For tumor staging, the Cancer staging system defined by the 7th Edition of the American Joint Committee was used. Besides, 40 pairs of HCC and respective adjacent non-tumorous samples were collected to analyze the relationship between FOXC2 and Ang-2.

### Animal, cell lines and cultures

Nude mice were purchased from Southern Medical University, Laboratory Animal Center. Normal hepatic cell line L02 and HCC cell lines including sk-hep-1, hep3B, SMMC-7721, Huh-7 and MHCC-97H were purchased from the Chinese Academy of Sciences, Shanghai Cell Bank (China). 293T cells were purchased from ATCC (American Tissue culture collection). All cells were maintained in RPMI1640 (HyClone, Logan, Utah, USA) containing 10% fetal bovine serum at 37 °C.

### Gene knockdowns through lentivirus-delivered shRNA

For the gene knockdown (KD) studies, shRNA containing lentiviral particles for either FOXC2 or Ang-2 were purchased from Santa Cruz Biotechnology (Cat. No: sc-43767-V and sc-39305-V). The virus was collected after transfection for 60 h into 293T cells with all vectors and then infected target cells along with 8 mg/mL polybrene (Sigma-Aldrich). Western blotting was performed for selection of stable and independent clones.

### Lentivirus-mediated gene expression

Lentiviral expression clones LV-pGV208-FOXC2, lentiviral packaging plasmids (pHelper 1.0 and 2.0) and control vector LV-pGV208 were purchased from Genechem (Shanghai, China). The supernatant containing lentivirus particles were harvested after transfection for 48 h in 293T cells and then were used to infect Huh-7 and MHCC-97H cells. Based on EGFP assay, lentiviral-infected cells were sorted with flow cytometry to achieve 100% cell infection. Overexpression of FOXC2 was confirmed through western blot.

### Western blot

The protein of each group was collected after cell lysis with RIPA buffer and quantified using a BCA assay kit from Abcam (Cambridge, MA, USA). 20 µg of protein was separated on 10% SDS-PAGE gel, electrotransferred to nitrocellulose membranes (Thermo Fisher, USA), blocked with 1% BSA for 1 h and incubated with rabbit primary antibodies in 1:1000 dilution, including anti-Ang-2(#2948S), anti-FOXC2(#12974S),anti-E-cadherin(#14472S), anti-vimentin(#5741S), anti-fibronectin(#4705S) and anti-β-actin(#4970S) (Cell Signaling Technologies,USA). Subsequently, the membranes were washed extensively with PBST (Tween-20, 0.1%) followed by incubation of secondary antibody, HRP-conjugated anti-rabbit IgG incubation for 1 h at room temperature. The ECL method was then used to visualize protein bands.

### Real-time RT-PCR analysis

Total RNA was extracted from HCC cells according to manufacturer’s instructions using Trizol (Invitrogen). 1 μg of RNA was used to synthesize cDNA with the SuperScript^®^ III First-Strand Synthesis System (Invitrogen). Real-time PCR was performed with a CFX96 Real-Time System (Bio-Rad) in the presence of 2X SYBR green master mixture (Invitrogen) in a final reaction volume of 10 μL. The primer sequences were: FOXC2 sense 5′-CCTACCTGAGCGAGCAGAAT-3′, antisense 5′-ACCTTGACGAAGCACTCGTT-3′, GAPDH sense 5′-TGTTGCCATCAATGACCCCTT-3′, and antisense 5′-CTCCACGACGTACTCAGCG-3′. The internal control was GAPDH. All experiments were run in triplicates in three independent experiments.

### CCK8 assay

CCK-8 (Yeasen, Shanghai, China) was used to assess the cell proliferation as manufacturer’s instructions recommend. Briefly, cells were inoculated in 96-well plates at the density of 1.5 × 103 cell/well, and cultured in incubator for 24 h before evaluated at day 1, 2, 3, 4, 5, respectively. CCK-8 solution was then dripped into each well, and the plate was transferred to the incubator for 4 h. Finally, OD value at 450 nm was detected by HBS-1096B microplate reader (Detie, Nanjing, China).

### Wound healing assay

The migration of cells was determined through wound healing tests following a standardized protocol. Briefly, 2 × 10^4^ cells were inoculated in each well of a 6-well plate and maintained for 24 h in an incubator. Next day, a 1 mL pipette tip was used to scratch the cells at a width of 500 μm and then washed with RPMI-1640. At 24 and 48 h after scratch, cells were observed with an inverted microscope and photograph. Data summarize three independent experiments.

### Cell invasion assay

Cells were incubated in PBS (phosphate-buffered saline) and the upper chambers coating with 50% Matrigel (BD Biosciences) of 24-well transwell plates (Corning Incorporated, NY, USA). After incubating for 24 h, invaded cells were examined and photographed through bright field microscopy (OLYMPUS cx31, TOKYO, Japan) after staining with 0.5% crystal violet. The invasion rate was determined by counting the invaded cells in five randomly selected fields of each chamber under a fluorescence microscope (OLYMPUS IX71, Tokyo, Japan). Data summarize three independent experiments.

### Immunohistochemistry(IHC)

Tissue sections were boiled in citrate-hydrochloric acid (pH 6.0) for 30 min for epitope retrieval and incubated at 4 °C overnight with anti-FOXC2 (Sigma, America) and anti-Ang-2 (Zsbio, China) primary antibodies. Immunostaining was performed using the Envision System with diaminobenzidine (Dako Cytomation, Glostrup, Denmark).

The stained sections were scored independently by two pathologists who were blinded to the clinical parameters. FOXC2 expression was evaluated using the extent and intensity of staining. The staining intensity was scored as 0-no staining, 1-weak staining(seen as light yellow), 2-moderate staining(seen as yellow–brown),or 3-strong staining(seen as brown).The extent of staining was scored as 0(0%), 1(1 to 25%), 3(51 to 75%) and 4(76 to 100%), based on the percentage of positive stain relative to the entire cancerous area or the entire section for normal samples. The final staining score (0 to 7) was defined through the sums of the intensity and extent scores. Tumors with a final staining score < 3 were considered as low expression, while those with high expression had scores > 3 to 7.

### Luciferase reporter assay

The binding regions of FOXC2 on Ang-2 promoter were predicted on the high-quality transcription factor binding profile database JASPAR [24]. Three segments of the Ang-2 promoter (Ang-2 promoter-WT:5′ -TGAGCCAACATTGCCCC —GAGTGAATATCCAA-3′, Ang-2 promoter-Mut1: 5′ -CAGTTACGTCTTGCCCCACTGC -3′, Ang-2 promoter-Mut2: 5′ -AGTCACGGCCCCAAGTGGAG-3′) were cloned into pGL3-basic-Report (Promega, WI, USA), as was human 3f-UTR of the FOXC2 gene. The reporter vectors containing Ang-2 and pGL3-FOXC2 promoter were transfected into MHCC-97H cells and luciferase activity was measured after 48 h using a dual-luciferase reporter assay system (Promega, WI, USA).

### In vivo tumor proliferation assay

FOXC2-OE Huh-7 and FOXC2-OE-Ang-2 KD Huh-7 cells were transfected to express luciferase. 6 to 7-week-old male NOD/SCID mice (Laboratory Animal Center of Southern Medical University, Guangzhou, China) were injected were with 2*10^6^ HCC cells in 100 µl 1:1 of PBS and matrigel on both sides of each mouse. The growth rate of the tumor was monitored using bioluminescent imaging. Mice were anesthetized with 0.7% pentobarbital sodium (10 μl/g) and injected intraperitoneally with 150 mg/kg luciferin (Caliper Life Sciences). Tumors were imaged through the IVIS Lumina II platform and analyzed with Live Image software (both from Caliper Life Sciences). Each group had six mice.

### Statistical analysis

All data are presented as mean ± SEM unless stated otherwise. Statistical significance (P < 0.05) was determined by two-side *t* test or ANOVA; The differences were assessed using SPSS 19.0 software (SPSS Inc., Chicago, IL). P ≤ 0.05 was considered as statistically significant.

## Results

### FOXC2 expression in hepatocellular carcinoma tissues

**Fig. 1:**
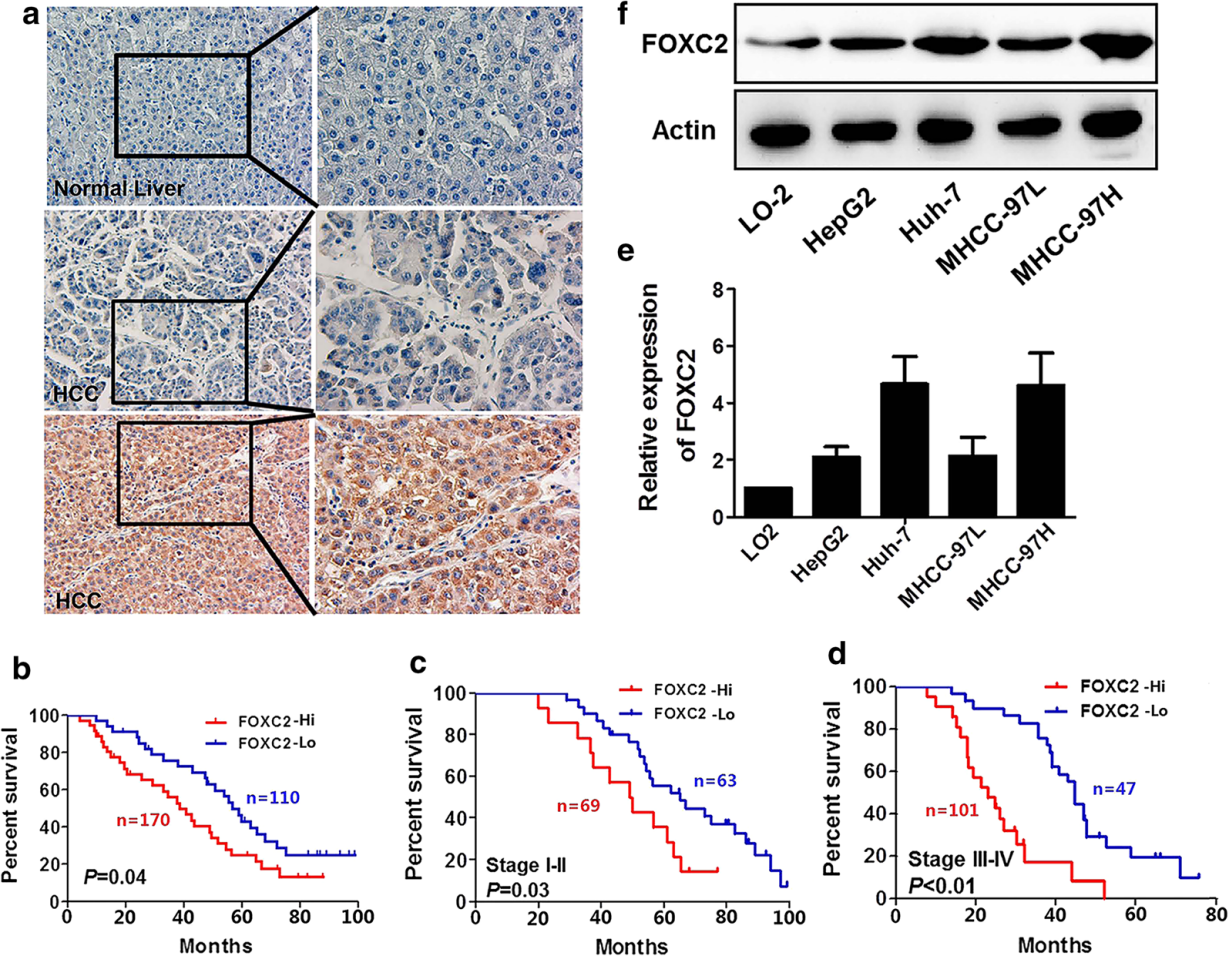
FOXC2 expression in HCC (hepatocellular carcinoma) tissues. **a** IHC assays to evaluate expression of FOXC2 in adjacent non-tumor tissues and tumor tissues. **b** OS was determined by Kaplan–Meier analysis as the FOXC2 low group versus the FOXC2 high group in the present study. **c** Kaplan–Meier analysis in TNM stage I-II for OS exhibited as the FOXC2 low group versus the FOXC2 high group. **d** OS was determined through Kaplan–Meier analysis and presented as the FOXC2 low group versus the FOXC2 high group in TNM stage III-IV. **e** The FOXC2 protein levels in four HCC cell lines and L02 cells were assesses through western blot. **f** QPCR to examine the FOXC2 mRNA levels in four HCC cell lines and L02 cells

280 paraffin-embedded HCC samples and 40 normal (non-cancer) samples were immunohistochemically analyzed for FOXC2 expression. According to the intensity of IHC staining, no or weak staining of FOXC2 protein was seen in 115 of 280 (41.1%) paraffin-embedded HCC tissues, while moderate staining (in the membrane and cytoplasm of cancer cells) was observed in 104 of 280 (37.1%) samples and strong staining was observed in 61 of 280 (21.8%) samples. In 40 non-cancerous control tissues, no FOXC2 staining was seen in 32 cases and weak expression was seen in two cases (Representative images in Fig. 1a). HCC cases were then divided into two groups according to the extent and intensity of FOXC2 staining: the low FOXC2 expression group (FOXC2-Lo) and the high FOXC2 expression group (FOXC2-Hi).

HCC patients in the FOXC2-Hi group showed more aggressive clinicopathologic characteristics, including higher serum AFP levels, larger tumor size, more recurrence, lower tumor differentiation, and later clinical stage (Table 1). Survival analysis demonstrated that the FOXC2-Hi group had significantly shorter overall survival (OS) compared to those in the FOXC2-Lo group (p = 0.04) (Fig. 1B). Multivariate survival analysis showed FOXC2 expression was an independent prognostic factor for HCC patients after radical resection (hazard ratio = 1.772, 95% confidence interval: 1.011 ~ 3.107, p = 0.045) (Table 2).

**Table 1:** Correlation between FOXC2 expression and clinicopathologic

Variable	N	FOXC2 expression		*P* value
		High	Low	
Gender				0.301
Male	220	130	90	
Female	60	40	20	
Age (years)				0.211
< 50	173	100	73	
≥ 50	107	70	37	
Tumor size (cm)^△^				0.000
<5	150	70	80	
≥ 5	130	100	30	
Serum HBsAg				0.582
Positive	245	147	98	
Negative	35	23	12	
Serum AFP(ng/ml)				0.460
< 25	157	92	65	
≥ 25	123	78	45	
Cirrhosis				0.806
Presence	120	74	46	
Absence	160	96	64	
UICC stage				0.007
I + II	132	69	63	
III + IV	148	101	47	
Metastasis/Recurrence				0.014
Yes	154	104	50	
No	126	66	60	
Edmondson grade				0.429
Low (I/II)	192	120	72	
High (III/IV)	88	50	38	

△: The largest dimension of the tumor specimen

**Table 2:** Cox regression analysis of patients with HCC

Variables	Univariate		*P* value
	HR	CI (95%)	
FOXC2 expression (1 = down, 2 = over)	1.772	1.011 ~ 3.107	0.045
Gender (1 = male, 2 = female)	0.433	0.365 ~ 1.034	0.102
Age (1< 50,2 ≥ 50)	0.813	0.341 ~ 1.532	0.352
Serum HBsAg (1 = negative, 2 = positive)	1.537	0.713 ~ 3.811	0.247
Serum AFP (1< 25 ng/ml, 2 ≥ 25 ng/ml)	1.953	1.046 ~ 3.900	0.021
Tumor size (1< 5 cm, 2 ≥ 5 cm)	2.015	1.334 ~ 3.822	0.040
Cirrhosis (1 = Absence, 2 = Presence)	1.151	0.541 ~ 2.388	0.157
Metastasis/Recurrence (1 = no, 2 = yes)	3.71	1.830 ~ 6.043	0.000
UICC stage (1 = I+II, 2 = III + IV)	2.225	1.019 ~ 4.023	0.025
Edmondson grade (1 = High (III/IV), 2 = Low (I/II))	0.911	0.562 ~ 1.831	0.238

### Effect of FOXC2 expression on HCC patient prognosis

This study included 75 patients with stage I, 57 with stage II, 82 with stage III, and 66 with stage IV HCC. We divided patients into two subgroups: early stage (TNM stage I-II) and late stage (stage III-IV). In the early stage group, 69 patients showed high FOXC2 expression in tumor cells. Patients with high FOXC2 expression suffered from worse OS as compared to those with low FOXC2 expression (P = 0.03, Fig. 1c). In the late stage group, the OS of the FOXC2-Lo group was longer than the FOXC2-Hi group (P < 0.01, Fig. 1d).

### FOXC2 expression in HCC cell lines

**Fig. 2:**
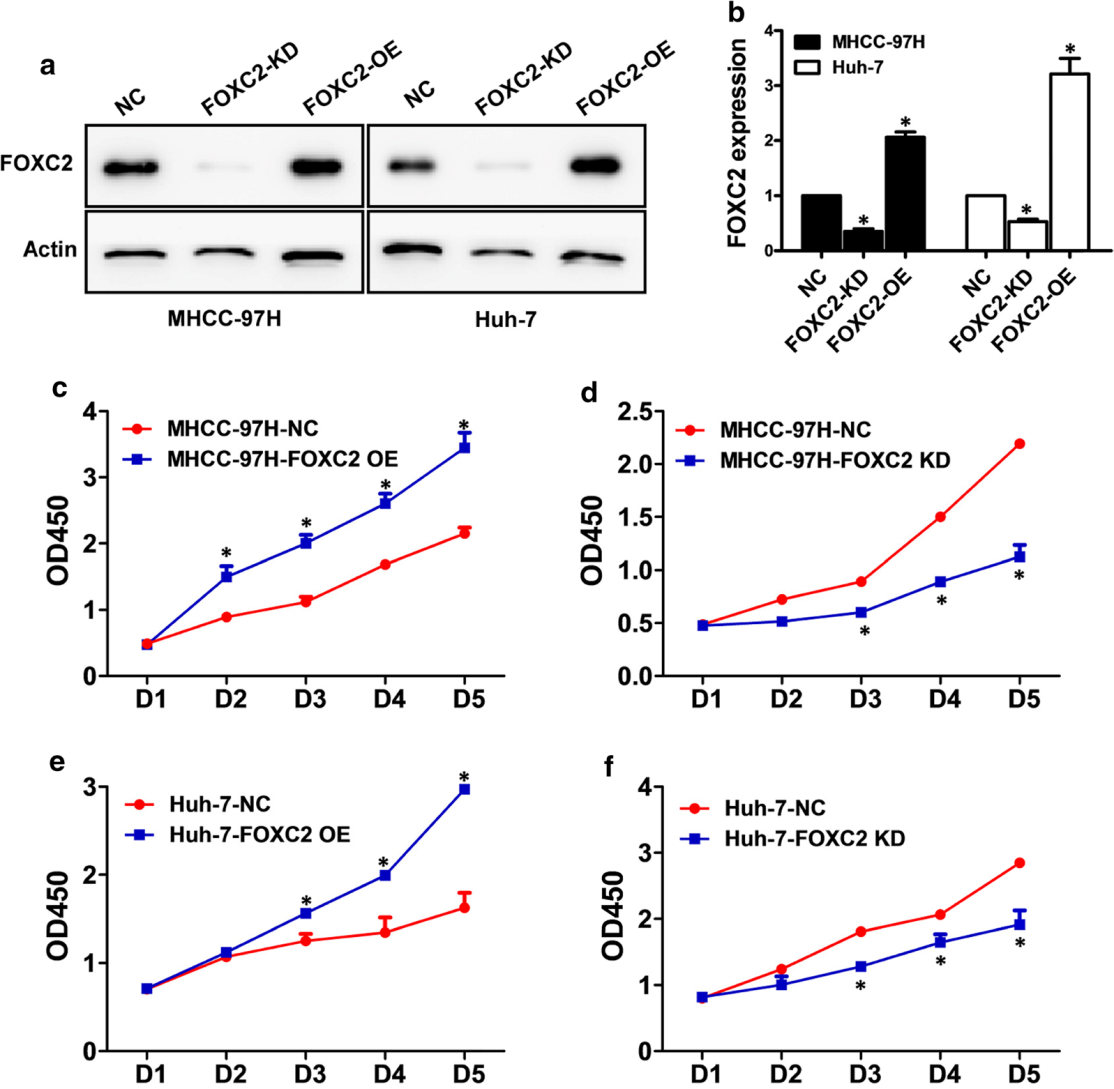
Knockdown and increased expression of FOXC2 in HCC MHCC-97H and Huh-7 cell lines. The expression of FOXC2 was detected by western blot and qPCR analysis in MHCC-97H and Huh-7 cells with FOXC2 KD (knockdown), OE (overexpression), or NC (negative control)(**a, b**). MTT assay to measure the cell growth of MHCC-97H (**c, d**) and Huh-7 (**e, f**) at various time points. Cell proliferation was affected by silencing or overexpressing FOXC2 when contrasted with negative controls. Significance at p < 0.01 is denoted by ‘*’ relative to control by student’s t-test

The levels of FOXC2 expression in different cell lines were measured with western blot and RT-PCR. HepG2, Huh-7, MHCC-97L and MHCC-97H all displayed higher expression levels of FOXC2 (Fig. 1e, f) than immortalized hepatocyte LO-2. FOXC2 was knocked down or overexpressed in the MHCC-97H and Huh-7 HCC cell lines to further investigate its potential role in HCC. Western blot and qPCR analysis were carried out to confirm the decrease and increase FOXC2 expression in the KD (knockdown) cell lines and OE (overexpression) cells, in comparison to their respective controls (Fig. 2a, b).

### FOXC2 promoted cell proliferation of HCC

Robust proliferative activity is vital for tumor metastasis and invasion. Here, we investigated changes in cell growth of FOXC2 knockdown (KD) and overexpression (OE) HCC cell lines using MTT assay. As shown in Fig. 2c and e, overexpression of FOXC2 promoted the proliferation of both MHCC-97H and Huh-7 cell (P < 0.01). In addition, the in vitro growth of MHCC-97H and Huh-7 cells was markedly inhibited after interference with FOXC2-shRNA (P < 0.01, Fig. 2d and f), thus indicating a positive correlation between FOXC2 expression and the cellular growth of HCC.

### FOXC2 promoted cell migration and invasion of liver cancer

**Fig. 3:**
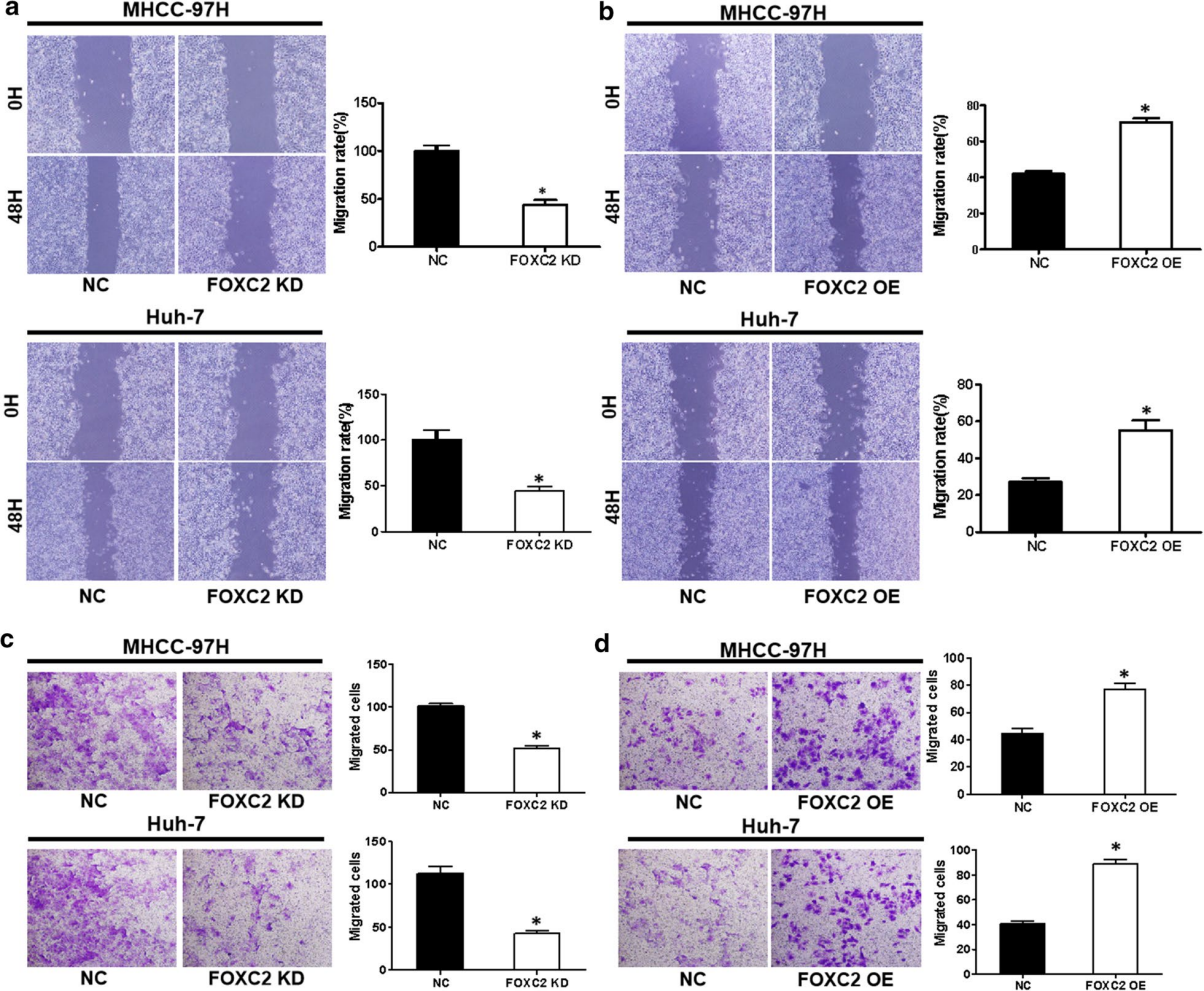
FOXC2 knockdown or overexpression and its effect on movement and invasion of MHCC-97H and Huh-7 cells. **a** Decrease of FOXC2 level in MHCC-97H and Huh-7 cells led to a significant decrease in healing of wound. **b** Increased FOXC2 level in MHCC-97H and Huh-7 cells led to a remarkable increase in healing of wound. **c** Reduced FOXC2 level in MHCC-97H and Huh-7 cells led to a significant reduction in invasive migration. **d** Enhanced FOXC2 level in MHCC-97H and Huh-7 cells led to a remarkable enhancement in invasive migration. Images represent of the outcomes of three experiments conducted independently. Values are presented as mean ± SEM. Significance at p < 0.01 is denoted by ‘*’ relative to control by student’s t-test

To analyze the impact of FOXC2 on the migration of both HCC cell lines, we performed wound-healing assays. As illustrated in Fig. 3a, b, FOXC2 knockdown led to an apparent reduction in wound healing at 48 h in both cell lines, while FOXC2 overexpression caused an noticeable improvement in migration capability (P < 0.01). In addition, transwell assays revealed that FOXC2 knockdown significantly reduced cell invasion, while additional FOXC2 yielded the opposite impact (P < 0.01, Fig. 3c, d).

### The expression of FOXC2 and Ang-2 in HCC tissues

**Fig. 4:**
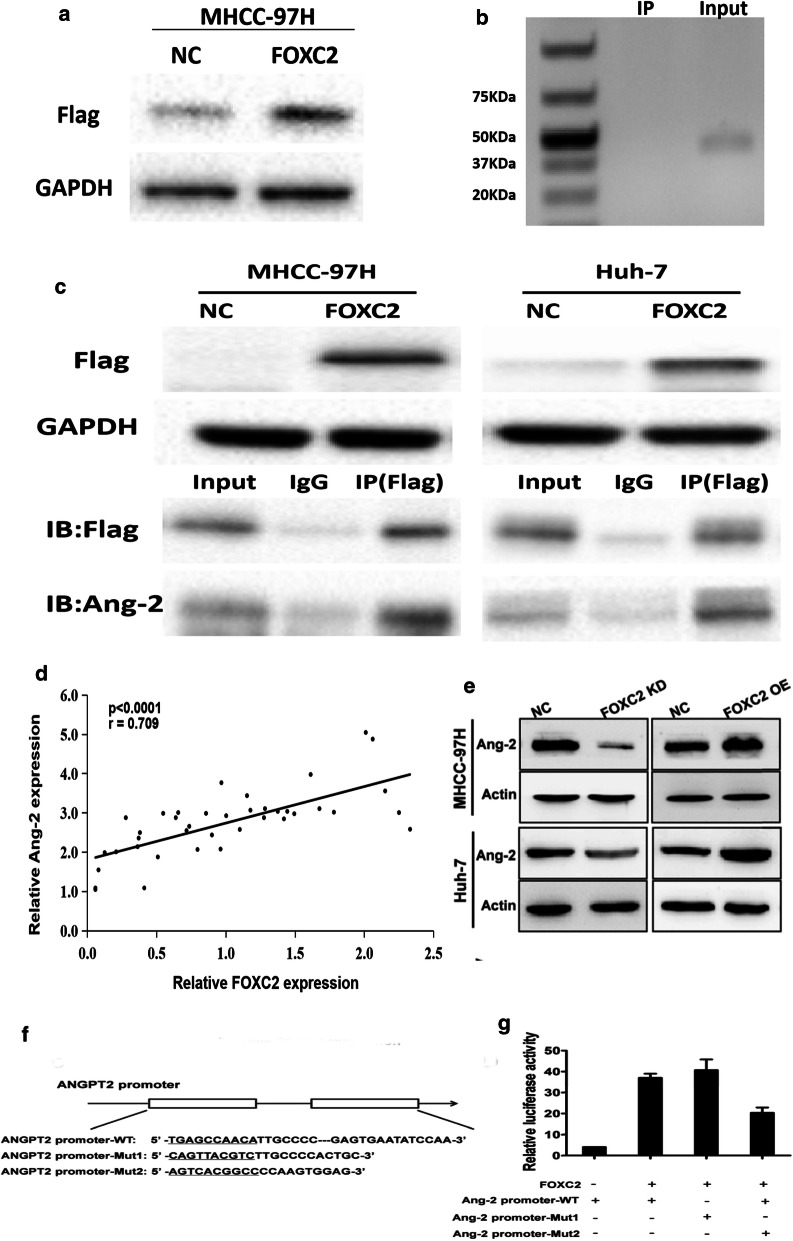
Correlation between expression of FOXC2 and Ang-2 in HCC tissues. **a** The correlation between FOXC2 and Ang-2 expression levels (R^2^ = 0.709, p < 0.0001) in terms of bivariate analysis. **b** The Ang-2 expression was detected through western blot assay on MHCC-97H and Huh-7 cells when FOXC2 was knocked-down and overexpressed; the membrane was probed with antibody to β-actin. **c** pGL3-FOXC2 was transfected into MHCC-97H cells that had been previously transfected with Ang-2 promoter-WT, Ang-2 promoter-Mut1, or Ang-2 promoter-Mut2 construct. **d** The activities of three Ang-2 promoters were determined through luciferase assay. The SD was obtained by conducting three experiments independently

According to the above results, we further analyzed FOXC2 and Ang-2 expression in 40 HCC tissues, of which 60.2% showed high FOXC2 expression. The result showed that tissues with high FOXC2 expression also exhibited high Ang-2 expression. As shown in Fig. 4a, Spearman’s correlation analysis indicated that FOXC2 expression was positively correlated with Ang-2 in HCC tissues (R^2^ = 0.709, p < 0.0001). Thus, FOXC2 may impact the production of Ang-2 to induce metastasis and invasion in HCC.

### FOXC2 mediated Ang-2 expression in HCC cells

Since Ang-2 also plays a crucial role in metastasis and invasion of malignancy, we further investigated whether FOXC2 modulates Ang-2 expression in MHCC-97H and Huh-7 cells via western blot. As shown in Fig. 4b, a reduction in Ang-2 expression was seen in cells with reduced FOXC2 level. Contrarily, cells with overexpressed FOXC2 exhibited enhanced Ang-2 expression, indicating that Ang-2 may act as a transcriptional target of FOXC2.

### FOXC2 activated the Ang-2 promoter

To determine whether Ang-2 is a direct target of FOXC2, three Ang-2 promoter fragments (putative mutant FOXC2 target site, or the wild type) were cloned into a pGL3-control vector for dual luciferase reporter assay. As observed in Fig. 4c, d, there was a decrease in luciferase activity in cells cotransfecting with both Ang-2 promoter-WT and pGL3-FOXC2 as compared to those with and pGL3-FOXC2 and Ang-2 promoter-Mut1/Mut2 or blank control. The results indicated that the Ang-2 promoter is directly targeted by FOXC2 for its transcriptional expression.

### FOXC2 facilitated migration and invasion of HCC cells through induction of Ang-2 expression

**Fig. 5:**
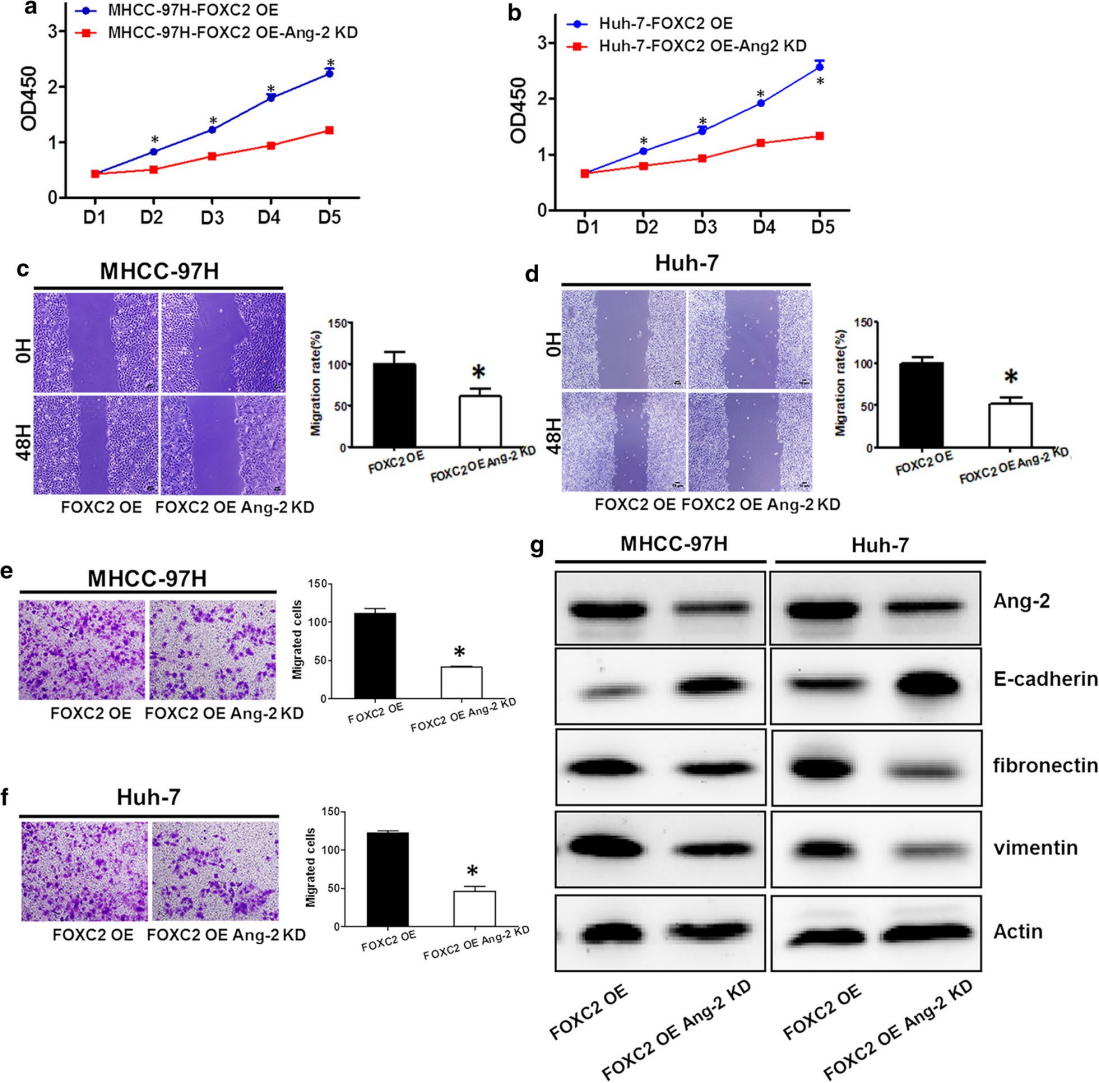
Knockdown of Ang-2 in MHCC-97H and Huh-7 cells with FOXC2 overexpression. Ang-2 knockdown led to impairment of cell proliferation of both MHCC-97H (**a**) and Huh-7 (**b**) cells with FOXC2 overexpression. Decreased Ang-2 contributed to impairment of cell migration of both cells with FOXC2 overexpression (**c–d**). The FOXC2 overexpressing cells with Ang-2 knockdown exhibited decreased cell invasion (**e–f**). **g** Western blot assessing Ang-2 levels in MHCC-97H and Huh-7 cells with FOXC2 overexpression and Ang-2 knockdown (KD). Significance at p < 0.01 is denoted by ‘*’ relative to control by student’s t-test

As expected, Ang-2 knockdown (KD) abated the growth of HCC cells with FOXC2 overexpression (OE) (Fig. 5a, b). As illustrated in Fig. 5c, d, Ang-2 downregulation rather than control significantly impaired the migratory capacity of both MHCC-97H and Huh-7 cells with FOXC2 overexpression. Further, the invasion of FOXC2 OE HCC cells was impaired with decreased Ang-2 expression (Fig. 5e, f). These findings confirmed that Ang-2 depletion abrogated FOXC2-induced cell proliferation and invasion.

### FOXC2 modulated EMT process of HCC cells

The function of Ang-2 knockdown on the FOXC2-induced EMT process in HCC was assessed by measuring the expression of E-cadherin, vimentin, and fibronectin in FOXC2 OE and FOXC2 OE Ang-2 KD cells through western blot. Reduced Ang-2 expression in FOXC2 OE cells led to the enhanced expression of epithelial marker E-cadherin and weakened the expression of mesenchymal markers fibronectin and vimentin (Fig. 5g). These findings thus implicated that FOXC2 may promote EMT process of HCC cells through the mediation of Ang-2.

### FOXC2 facilitated tumor growth in vivo

The aforementioned in vitro findings strongly indicated that FOXC2 promoted the proliferation and invasion of HCC cells by modulating Ang-2 expression. We next evaluated the effect of FOXC2 on tumor growth by in vivo optical imaging in NOD/SCID mice.

**Fig. 6:**
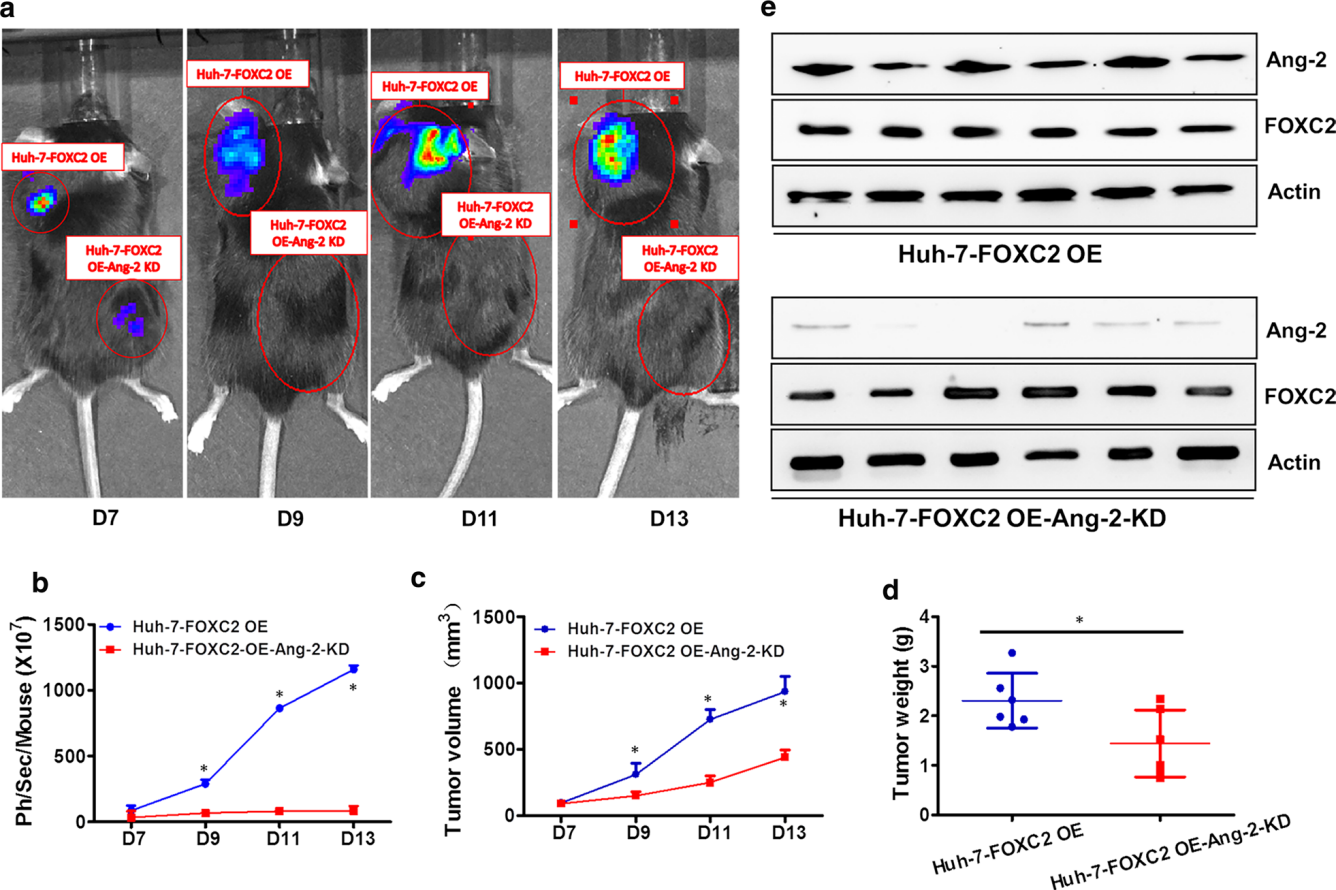
FOXC2 facilitates in vivo and in vitro proliferation of HCC through Ang-2. **a** Representative images of NOD/SCID mice injected with FOXC2 OE and FOXC2 OE Ang-2 KD Huh-7 cells at day7, 9, 11 and 13. **b** Quantification analysis of bioluminescent signal of tumor-bearing mice injected with FOXC2 OE or FOXC2 OE Ang-2 KD Huh-7 cells at specified time points. Ang-2 downregulation impeded proliferation of FOXC2 OE Huh-7 cells **c** Tumor volume of FOXC2 OE Huh-7 cells were decreased with Ang-2 downregulation. **d** Tumor weight of FOXC2 OE Huh-7 cells were lightened with Ang-2 knockdown. **e** Ang-2 and FOXC2 expression in FOXC2 OE and FOXC2 OE Ang-2 KD tumors were analyzed through western blot. Values are presented as mean ± SEM. Significance at p < 0.01 is denoted by ‘*’ relative to control by one-way ANOVA

FOXC2 OE and FOXC2 OE Ang-2 KD Huh-7 cells with luciferase activity were injected into the flanks and shoulders of NOD/SCID mice (Fig. 6a and e). A significant reduction in bioluminescence was found in mice injected with FOXC2 OE Ang-2 KD Huh-7 cells (Fig. 6b), compared with those injected with FOXC2 OE Huh-7 cells (P < 0.01). The enhanced expression of epithelial marker E-cadherin and reduced expression of mesenchymal markers fibronectin and vimentin were detected in FOXC2 OE Ang-2 KD group compared to FOXC2 OE group (Additional file 1: Figure S1). Furthermore, the tumor size and weight in the FOXC2 OE Ang-2 KD group were significantly reduced (P < 0.01, Fig. 6c, d), indicating that FOXC2 accelerates growth and metastasis of HCC through regulation of Ang-2.

## Discussion

By far, recurrence and metastasis remain the major cause of HCC-related mortality, and the underlying molecular mechanisms are still to be clarified.

As a focus of current research field, FOXC2 has been reported as an oncogene in a range of cancers. It was well accepted that FOXC2 was a metastasis-related gene and might consequently serve as a predictor for the treatment response or prognosis of cancer patients in clinic use [25]. In line with above views, we observed frequent and aberrant FOXC2 expression in HCC tissues and a significant positive correlation between FOXC2 expression and malignant clinicopathology of HCC patients including tumor size, UICC stage and metastasis/recurrence status. Moreover, FOXC2 expression was proved to be an independent risk factor in HCC patients treated with radical surgery. Furthermore, regardless of TNM stage, patients with high FOXC2 expression was associated with poorer OS than those with low FOXC2 expression, suggesting that FOXC2 expression may help to evaluate the outcome of HCC patients. In vitro experiments showed that the upregulation of FOXC2 promoted growth, motility and invasion of HCC cells, while decreased FOXC2 expression exhibited the opposite effects, indicating that FOXC2 might serve to facilitate the metastasis of HCC.

Further analysis revealed a positive correlation between the expression of FOXC2 and Ang-2, led to our assumption that FOXC2 promotes Ang-2 expression for the invasion and metastasis of HCC. Luciferase assay showed that FOXC2 noticeably augmented the transcriptional activity of Ang-2, together with the result that Ang-2 expression was decreased in FOXC2-knockdown MHCC-97H and Huh-7 cell lines, suggesting that Ang-2 was a direct target of FOXC2.

Hypervascularity is one of the remarkable features of HCC. In the past decades, the development of neovascular targeting therapy has dawned on HCC patients. The current therapeutic target of the angiogenic pathway includes VEGF signaling, angiopoietin 1(Ang-1), Ang-2 and tyrosine-protein kinase receptor Tie2. Agents targeting VEGF axis like bevacizumab, axitinib, lenvatinib, pazopanib, ramucirumab, sunitinib and vandetanib has been approved for the treatment of various cancers including colorectal cancer, non-small cell lung cancer, cervical cancer,ovarian cancer. Angiogenesis inhibitor targeting both VEGFR and Tie2 like cabozantinib, regorafenib and sorafenib have also shown efficacy in the treatment of renal cancer, thyroid cancer, gastrointestinal stromal tumor and liver cancer [26]. Among them, sorafinib is the first approved agent for the treatment of advanced HCC and lenvatinib is the first agent that is non-inferior to sorafenib during the ten years’ development of targeted therapy for HCC [27]. Ang-2 not only correlates with malignant phenotype and prognosis of HCC, but also facilitates VEGF-mediated neovascularization in HCC [23]. Moreover, the fact that Ang-2 expression is co-expressed with VEGF in HCC and connected to worse survival of HCC patients treated with sorafenib indicated that Ang-2 may contribute to the resistance of anti-VEGF therapy [21, 28]. Recently, Ang-2 has also been implicated as an important proangiogenic factor in the inflammatory processes and was found to be upregulated in various signaling pathways and inflammation-related tumors [20, 29]. Abnormal Ang-2 expression was shown to increase the generation and permeability of blood vessels in both the ischemic and hypoxic environment [30]. Intratumoral hypoxia induced Ang-2 expression with increasing tumor volume to promote angiogenesis and metastasis [31]. In addition, Ang-2 accelerated cell proliferation and altered the EMT process, invasion, and metastasis of lung cancers [32]. Here, we observed that decreased Ang-2 expression impaired FOXC2-induced migration and invasion, which was accompanied by reduced E-cadherin and enhanced vimentin and fibronectin expressions in HCC cells with FOXC2 overexpression, indicating that FOXC2 may facilitate the EMT process in HCC via regulation of Ang-2. In vivo experiments further supported this conclusion by showing that Ang-2 downregulation diminished tumor growth, volume and size of HCC.

## Conclusions

To sum up, this study indicated that FOXC2 promote HCC migration and invasion, and the regulation of Ang-2 exacerbates these effects. Thus, FOXC2 and Ang-2 might be potent and promising targets for the prevention and treatment of metastatic HCC.

## Supplementary information

**Additional file 1: Figure S1.** Fibronectin, vimentin and E-cadherin expression in FOXC2 OE and FOXC2 OE Ang-2 KD tumors were analyzed through western blot.

## Data Availability

The datasets analyzed and/or used in this current study can be obtained by requesting the corresponding author.

## Abbreviations

FOXC2: Forkhead box C2
Ang-2: Angiopoietin-2
HCC: Hepatocellular carcinoma
ATCC: American Tissue culture collection

## References

[1] Asrani SK, Devarbhavi H, Eaton J, Kamath PS (2019). Burden of liver diseases in the world. J Hepatol.

[2] Sia D, Villanueva A, Friedman SL, Llovet JM (2017). Liver cancer cell of origin, molecular class, and effects on patient prognosis. Gastroenterology.

[3] Shiani A, Narayanan S, Pena L, Friedman M (2017). The role of diagnosis and treatment of underlying liver disease for the prognosis of primary liver cancer. Cancer Control.

[4] Díaz-González Á, Reig M, Bruix J (2016). Treatment of hepatocellular carcinoma. Digest Dis.

[5] Golson ML, Kaestner KH (2016). Fox transcription factors: from development to disease. Development.

[6] Wang J, Li W, Zhao Y, Kang D, Fu W, Zheng X, Pang X, Du G (2018). Members of FOX family could be drug targets of cancers. Pharmacol Ther.

[7] Zaiss DM, Coffer PJ (2018). Forkhead box transcription factors as context-dependent regulators of lymphocyte homeostasis. Nat Rev Immunol.

[8] Werden SJ, Sphyris N, Sarkar TR, Paranjape AN, Labaff AM, Taube JH (2016). Phosphorylation of serine 367 of FOXC2 by p38 regulates ZEB1 and breast cancer metastasis, without impacting primary tumor growth. Oncogene.

[9] Li Q, Wu J, Wei P, Xu Y, Zhuo C, Wang Y (2015). Overexpression of forkhead Box C2 promotes tumor metastasis and indicates poor prognosis in colon cancer via regulating epithelial-mesenchymal transition. Am J Cancer Res.

[10] Nishida N, Mimori K, Yokobori T, Sudo T, Tanaka F, Shibata K (2011). FOXC2 is a novel prognostic factor in human esophageal squamous cell carcinoma. Annals surgical Oncol.

[11] Soleimani F, Hajjari M, Mohammad SB, Behmanesh MJCJ. Up-Regulation of FOXC2 and FOXQ1 Is Associated with The Progression of Gastric-Type Adenocarcinoma. 2017;19(Suppl 1):66-71. 10.22074/cellj.2017.4357 PMC5448320 28580309

[12] Wei J, Fan H, Cheng Q, Ding J, Wang Q, Pang XJBC. Prognostic value of high FoxC2 expression in resectable non-small cell lung cancer, alone or in combination with E-cadherin expression. 2016;16(1):16. 10.1186/s12885-016-2056-0 PMC4711004 26758745

[13] Cai J, Tian AX, Wang QS, Kong PZ, Du X, Li XQ (2015). FOXF2 suppresses the FOXC2-mediated epithelial-mesenchymal transition and multidrug resistance of basal-like breast cancer. Cancer Lett.

[14] Zhou Z, Zhang L, Xie B, Wang X, Yang X, Ding N (2015). FOXC2 promotes chemoresistance in nasopharyngeal carcinomas via induction of epithelial mesenchymal transition. Cancer Lett.

[15] Li C, Ding H, Tian J, Wu L, Wang Y, Xing Y (2016). Forkhead Box Protein C2 (FOXC2) promotes the resistance of human ovarian cancer cells to cisplatin in vitro and in vivo. Cell Physiol Biochem.

[16] Zhang CL, Zhu KP, Ma XL (2017). Antisense lncRNA FOXC2-AS1 promotes doxorubicin resistance in osteosarcoma by increasing the expression of FOXC2. Cancer Lett.

[17] Yang F, Lv L, Zhang K, Cai Q, Liu J, Jiang YJCP (2017). Elevated FOXC2 expression promotes invasion of HCC cell lines and is associated with poor prognosis in hepatocellular carcinoma. Cell Physiol Biochem.

[18] Muto J, Shirabe K, Sugimachi K, Maehara Y (2015). Review of angiogenesis in hepatocellular carcinoma. Hepatol Res.

[19] Jain RK (2003). Molecular regulation of vessel maturation. Nat Med.

[20] Alexander S, Plate KH, Yvonne RJ (2015). Angiopoietin-2: a multifaceted cytokine that functions in both angiogenesis and inflammation. Ann NY Acad Sci.

[21] Moon WS, Rhyu KH, Kang MJ, Lee DG, Yu HC, Yeum JH (2003). Overexpression of VEGF and angiopoietin 2: a key to high vascularity of hepatocellular carcinoma?. Mod Pathol.

[22] Scholz A, Rehm VA, Rieke S, Derkow K, Schulz P, Neumann K (2007). Angiopoietin-2 serum levels are elevated in patients with liver cirrhosis and hepatocellular carcinoma. Am J Gastroenterol.

[23] Bupathi M, Kaseb A, Meric-Bernstam F, Naing A (2015). Hepatocellular carcinoma: Where there is unmet need. Mol Oncol.

[24] Khan A, Fornes O, Stigliani A, Gheorghe M, Castro-Mondragon JA, Van dLR, et al. JASPAR 2018: update of the open-access database of transcription factor binding profiles and its web framework. 2017;77(21):e43.

[25] Wang T, Zheng L, Wang Q, Hu YW (2018). Emerging roles and mechanisms of FOXC2 in cancer. Clin Chim Acta.

[26] Fukumura D, Kloepper J, Amoozgar Z, Duda DG, Jain RK (2018). Enhancing cancer immunotherapy using antiangiogenics: opportunities and challenges. Nat Rev Clin Oncol.

[27] Llovet JM, Montal R, Sia D, Finn RS (2018). Molecular therapies and precision medicine for hepatocellular carcinoma. Nat Rev Clin Oncol.

[28] Llovet JM, Peña CE, Lathia CD, Shan M, Meinhardt G, Bruix J (2012). Plasma biomarkers as predictors of outcome in patients with advanced hepatocellular carcinoma. Clin Cancer Res.

[29] Biel NM, Siemann DW (2016). Targeting the Angiopoietin-2/Tie-2 axis in conjunction with VEGF signal interference. Cancer Lett.

[30] Wang Q, Lash GE (2017). Angiopoietin 2 in placentation and tumor biology: the yin and yang of vascular biology. Placenta.

[31] Keskin D, Kim J, Cooke VG, Wu CC, Sugimoto H, Gu C (2015). Targeting vascular pericytes in hypoxic tumors increases lung metastasis via angiopoietin-2. Cell Rep.

[32] Dong Z, Chen J, Yang X, Zheng W, Wang L, Fang M (2018). Ang-2 promotes lung cancer metastasis by increasing epithelial-mesenchymal transition. Oncotarget.

