# Supplementary figures and images for: FOXC2 is a prognostic biomarker and contributes to the growth and invasion of human hepatocellular carcinoma
Source: Cancer Cell Int. 2020 May 26;20:196. doi: 10.1186/s12935-020-01265-0 (PMC7249675; doi:10.1186/s12935-020-01265-0)

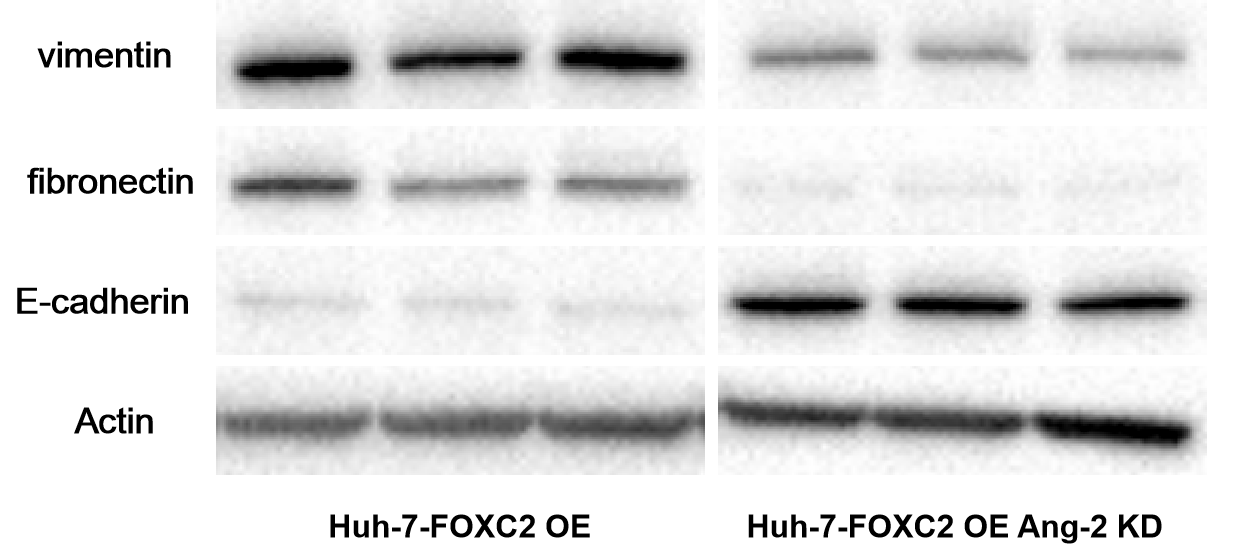

Supplement: Supplementary file 1 — Additional file 1: Figure S1. Fibronectin, vimentin and E-cadherin expression in FOXC2 OE and FOXC2 OE Ang-2 KD tumors were analyzed through western blot. [file 12935_2020_1265_MOESM1_ESM.tif]
